# Hyperosmosis and its combination with nutrient-limitation are novel environmental stressors for induction of triacylglycerol accumulation in cells of *Chlorella kessleri*

**DOI:** 10.1038/srep25825

**Published:** 2016-05-17

**Authors:** Kazuho Hirai, Taihei Hayashi, Yuri Hasegawa, Atsushi Sato, Mikio Tsuzuki, Norihiro Sato

**Affiliations:** 1 School of Life Sciences, Tokyo University of Pharmacy and Life Sciences, Horinouchi, Hachioji, Tokyo 192-0392, Japan; 2 JST, Chiyoda-ku, Tokyo 102-0075, Japan

## Abstract

Triacylglycerols of oleaginous algae are promising for production of food oils and biodiesel fuel. Air-drying of cells induces triacylglycerol accumulation in a freshwater green alga, *Chlorella kessleri*, therefore, it seems that dehydration, i.e., intracellular hyperosmosis, and/or nutrient-limitation are key stressors. We explored this possibility in liquid-culturing *C. kessleri* cells. Strong hyperosmosis with 0.9 M sorbitol or 0.45 M NaCl for two days caused cells to increase the triacylglycerol content in total lipids from 1.5 to 48.5 and 75.3 mol%, respectively, on a fatty acid basis, whereas nutrient-limitation caused its accumulation to 41.4 mol%. Even weak hyperosmosis with 0.3 M sorbitol or 0.15 M NaCl, when nutrient-limitation was simultaneously imposed, induced triacylglycerol accumulation to 61.9 and 65.7 mol%, respectively. Furthermore, culturing in three-fold diluted seawater, the chemical composition of which resembled that of the medium for the combinatory stress, enabled the cells to accumulate triacylglycerol up to 24.7 weight% of dry cells in only three days. Consequently, it was found that hyperosmosis is a novel stressor for triacylglycerol accumulation, and that weak hyperosmosis, together with nutrient-limitation, exerts a strong stimulating effect on triacylglycerol accumulation. A similar combinatory stress would contribute to the triacylglycerol accumulation in air-dried *C. kessleri* cells.

Triacylglycerols (TG) are esters constructed from one glycerol and three fatty acids, and are localized in intracellular lipid droplets as a major lipid component in eukaryotic and a part of prokaryotic organisms^1^. It is well known that TG are stored according to the life cycle program in oleaginous seeds of plants or to metabolic necessity in adipose tissues of animals^1^. Upon physiological demand, fatty acids are released from TG by lipases, and then subjected to ß-oxidation for the synthesis of acetyl-CoA^2^. Acetyl-CoA in general is utilized for the synthesis of energy-rich chemical compounds including ATP and NADH, or, in oleaginous seeds of plants, could be converted into higher molecular compounds through glyconeogenesis via the glyoxylate cycle^3^. Otherwise, the released fatty acids could be reassembled into membrane lipids as in bud formation in yeasts^4^. Accumulation of TG therefore is considered to be a strategy that ensures energy and reduced carbon for future use.

Distinct from seed plants and animals that possess special TG-storing organs or tissues, mono-cellular microalgae accumulate TG in single cells when they are subjected to aberrant growth conditions^5^. Nitrogen (N)-starvation is more effective for induction of TG accumulation in a wide range of algal species than any other condition including illumination at a high-light intensity, weak saline stress, or Fe ion depletion^5^,^6^,^7^,^8^. We recently reported two novel environmental stressors, i.e., sulfur (S)-starvation and air-drying of cells, for induction of TG accumulation in freshwater green algae, *C. kessleri* and *C. reinhardtii*^9^,^10^, consequently revealing that air-drying was as effective as N-starvation in *C. kessleri*^9^. The cells were placed on a glass fibre membrane for imposition of the air-drying stress, thereby being subjected to dehydration and limited availability to all nutrients^9^. Meanwhile, in N- or S-starved *C. reinhardtii* cells, photosynthesis can supply reduced carbon and high-chemical energy compounds, and is indispensable for TG synthesis^10^,^11^. In turn, TG synthesis contributes to the survival of N-starved *C. reinhardtii* cells through consumption of reducing power that is produced by ongoing photosynthesis, otherwise, reactive oxygen species (ROS) will be generated in excess, lowering cellular viability^12^.

Industrially, TG extracted from crop seeds has long been used for production of food oil, and, from a carbon-neutral aspect, has recently attracted attention as a material for biodiesel fuel^13^. At present, palm oil production is the greatest in the world, which, however, is based on destruction of the tropical rain forest for a large-scale plantation of palm trees^13^. As to biodiesel fuel, excessive production using crops would cause soaring food prices due to competition between biodiesel fuel and food production^13^. Algal cells can be cultured in appropriate apparatuses even on infertile land and would therefore preclude environmental destruction or competition with production of food crops^5^. The air-drying protocol, beside its effect of strongly inducing TG accumulation, has two merits from an industrial aspect: one is mitigating of the demand for electric power for drying of harvested cells that is unavoidable on lipid extraction, whereas the other, distinct from the classical N-starvation, needs no labor for culture medium exchange^9^. However, the biological mechanism underlying TG accumulation in air-drying cells, including identification of environmental stressor(s), has yet to be elucidated.

In the present study, we attempted to identify environmental factors for induction of TG accumulation under air-drying conditions, and thereby to discover novel stressor(s) for TG accumulation. For this purpose, the respective effectiveness of hyperosmosis, limitation of all nutrients, and their combination was investigated for TG accumulation in liquid-culturing cells of *C. kessleri*. Besides, on the basis of the obtained results, we could develop a novel and simple culturing system for TG accumulation in *C. kessleri* with the use of seawater.

## Results

### Effects of hyperosmotic stress on the TG content in *C. kessleri*

We previously suggested that dehydration and/or limitation of all nutrients could be stressors that induce accumulation of TG in air-drying cells of *C. kessleri*^9^. Here, the effect of dehydration in *C. kessleri* was evaluated by imposing hyperosmotic stress on liquid-culturing cells of it. The hyperosmotic stress imposed was weak (0.3 M sorbitol or 0.15 M NaCl), moderate (0.6 M sorbitol or 0.3 M NaCl), or strong (0.9 M sorbitol or 0.45 M NaCl). Since the TG content relative to total lipids (TL) on a fatty acid basis increased to a plateau level in a day in our culturing system (see below, e.g., its increase in cells cultured in 3-fold diluted artificial seawater), quantitative analysis of TG was performed with cells cultured for two days under the respective stress conditions.

**Figure 1:**
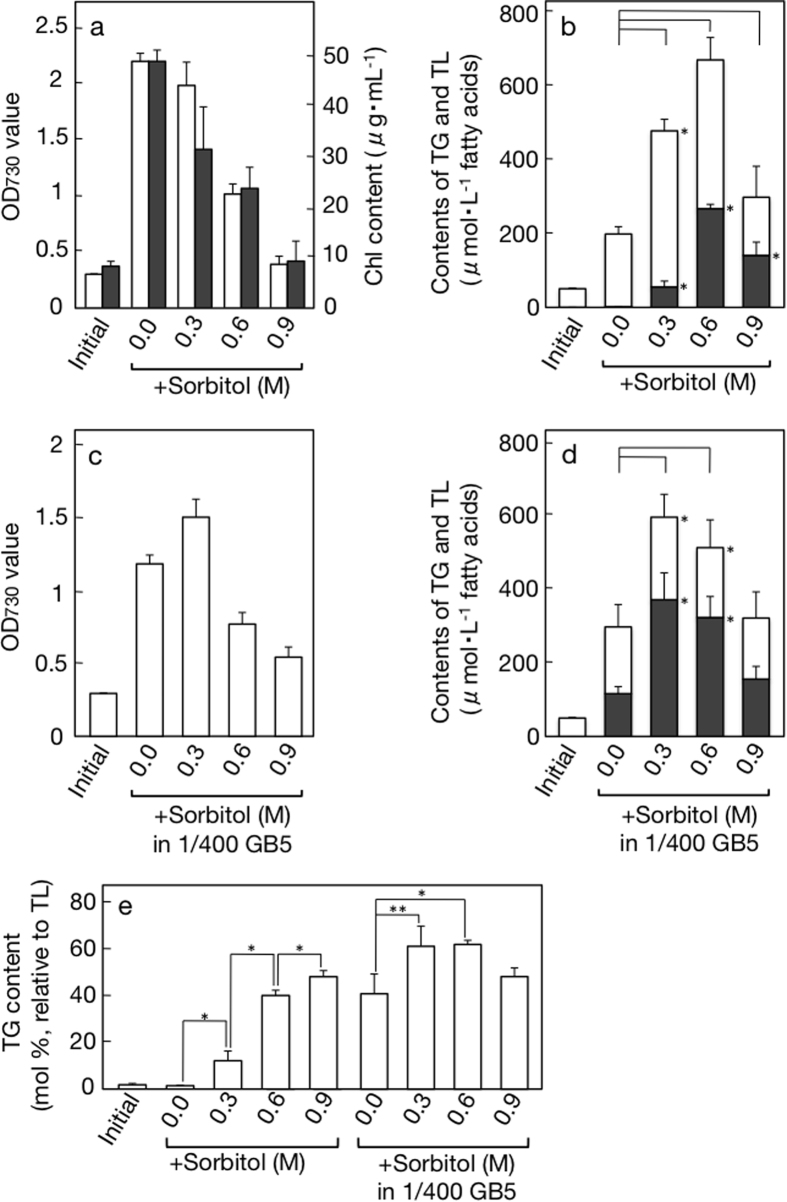
Effects of sorbitol-induced hyperosmotic, nutrient-limiting or combinatory stress on cell growth and TG accumulation in *C. kessleri*. The cells were cultured for 2 days under hyperosmotic conditions for measurement of (**a**) OD_730_ values (open bars) or Chl contents (closed bars), and (**b**) the contents of TG (closed bars), TL (see top values of open bars), and therefore polar lipids (open bars) in the cultures. The cells were cultured for 2 days under nutrient-limiting or combinatory stress conditions for measurement of (**c**) OD_730_ values and (**d**) the contents of TG (closed bars) and TL (see top values of open bars) in the cultures. (**e**) TG contents relative to TL, on the basis of fatty acids, estimated from data of (**b**) or (**d**). The values shown are the averages ± SE for three distinct groups of data. ‘Initial’ in (**a–e**) indicates the initial level. The initial levels of OD_730_ were adjusted to 0.3 for (**a,c**) whereas those of TG or TL are the same for (**b,d**). The significance of differences was evaluated by Student’s t test. *P < 0.05. **P < 0.1. In (**b,d**), see differences in the TG or TL content between 0.0, and 0.3, 0.6 or 0.9.

As regards cell growth (Fig. 1a), the OD_730_ value of the culture became 7.3-fold in two days under normal conditions. Inclusion of 0.3 M sorbitol slightly decreased the value to 6.6, thus causing 10% repression. When the sorbitol concentration was increased to 0.6 and 0.9 M, the extent of repression as to OD_730_ increased to 54 and 82%, respectively. The accumulated level of Chl, which is exclusively bound to the PSI and II complexes, the major protein complexes in thylakoid memberanes, also became lower with harsher hyperosmotic stress (Fig. 1a). The lowered OD_730_ values would thus reflect a repressed increase in biomass. Subsequent analysis of lipids demonstrated hyperosmosis-stimulated accumulation of TL in the culture on the basis of fatty acids (Fig. 1b, e.g., a 3.4-fold higher content of TL in 0.6 M sorbitol, relative to control in 0.0 M sorbitol). Concomitantly, the fatty acid content of TG amounted to only 2.9 μM under normal conditions, whereas it became higher to reach 56, 266, and 140 μM with 0.3, 0.6, and 0.9 M sobitol, respectively. The highest TG content in TL on a basis of fatty acid (48.5 mol%), however, was achieved not with 0.6 M, but with 0.9 M sorbitol (Fig. 1e). The most abundant accumulation of TG in the culture with 0.6 M sorbitol was due to its effect to less severely repress cell growth. Interestingly, despite the retarded increase in the Chl content and/or OD_730_ value in cells stressed with 0.3 or 0.6 M sobitol, the content of polar lipids was higher in the stressed cells than in control ones, which implicated activation of polar lipid synthesis by weak or moderate hyperosmosis (see open bars in Fig. 1b).

**Figure 2:**
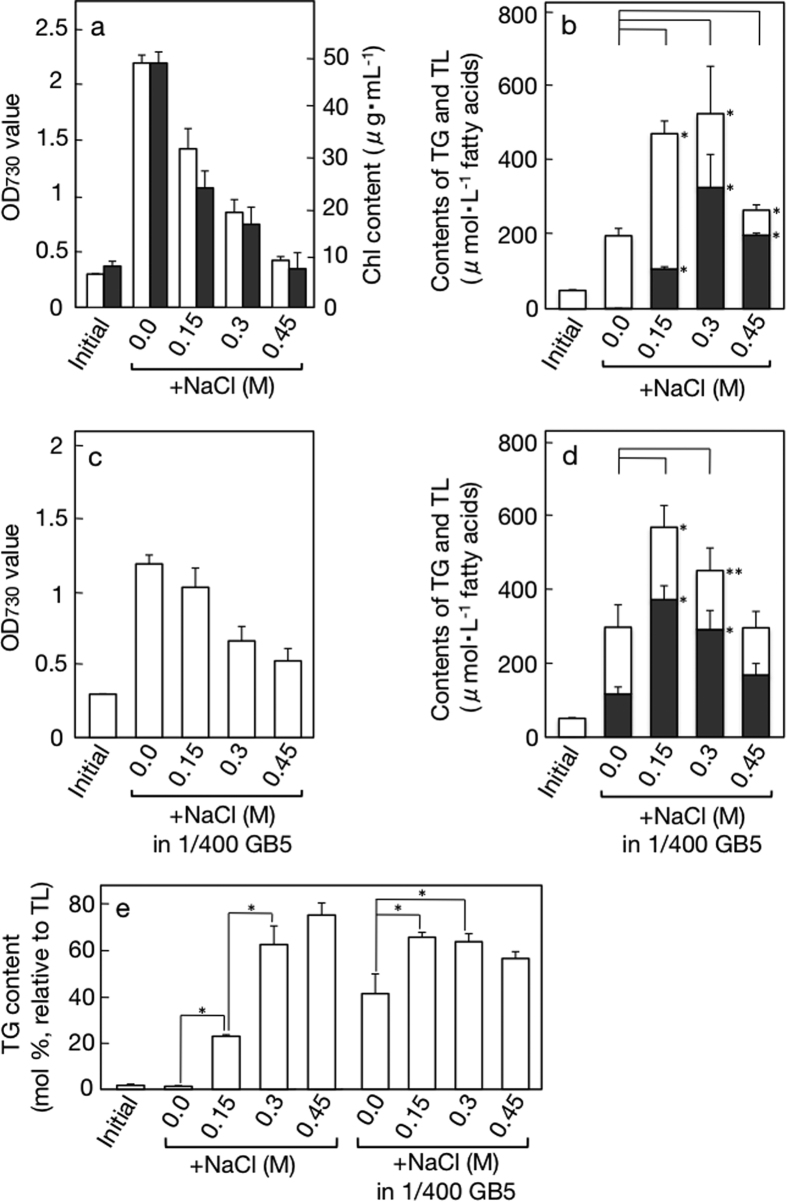
Effects of NaCl-induced hyperosmotic, nutrient-limiting or combinatory stress on cell growth and TG accumulation in *C. kessleri*. The cells were cultured for 2 days under hyperosmotic conditions for measurement of (**a**) OD_730_ values (open bars) or Chl contents (closed bars), and (**b**) the contents of TG (closed bars), TL (see top values of open bars), and therefore polar lipids (open bars) in the cultures. The cells were cultured for 2 days under nutrient-limiting or combinatory stress conditions for measurement of (**c**) OD_730_ values and (**d**) the contents of TG (closed bars) and TL (see top values of open bars) in the cultures. (**e**) TG contents relative to TL, on the basis of fatty acids, estimated from data of (**b**) or (**d**). The values shown are the averages ± SE for three distinct groups of data. ‘Initial’ in (**a–e**) indicates the initial level. The values shown are the averages ± SE for three distinct groups of data. The values of ‘initial’ and control are the same as those in Fig. 1. The significance of differences was evaluated by Student’s t test. *P < 0.05. **P < 0.1. In (**b,d**), see differences in the TG or TL content between 0.0, and 0.3, 0.6 or 0.9.

Meanwhile, NaCl, similar to sorbitol, retarded the increase of OD_730_ or Chl in a dose-dependent manner (Fig. 2a), however, the effect was more prominent than that of sorbitol for the weak and moderate hyperosmosis, respectively. The hyperosmotic stress with NaCl induced accumulation of TL and TG in the culture, with the moderate stress of 0.3 M NaCl, similar to that of 0.6 M sorbitol, exerting the greatest effect on TG to achieve accumulation to 327 μM (Fig. 2b). The TG content relative to TL was the highest at 0.45 M NaCl to account for 75.3 mol% (Fig. 2e). It was of particular note that NaCl, as compared with sorbitol, had a more pronounced impact on the accumulated level of TG at the respective hyperosmotic levels (Fig. 2b,e, cf., Fig. 1b,e). Concerning polar lipids, their synthesis seemed to be stimulated by 0.15 M NaCl (see open bars in Fig. 2b). Collectively, it was proved that hyperosmosis is a novel environmental stressor for induction of TG accumulation in *C. kessleri*. It is highly probable that the air-drying stress causes TG accumulation at least partially through the action of dehydration-induced intracellular hyperosmosis.

### Effects of nutrient-limiting stress on the TG content in *C. kessleri*

For air-drying stress imposition, *C. kessleri* cells are placed on a glass fibre membrane at a 100-fold higher cell density relative to the liquid culture, and thus there should have been intensified competition for acquisition of all nutrients included in the residual medium (1/4 GB5)^9^. We then examined the effects of limitation of all nutrients on TG accumulation in *C. kessleri* by culturing the cells in 100-fold diluted medium (1/400 GB5). The 1/400 GB5 medium contained 5.5, 67, and 0.25 μM S-, N-, and Fe-sources, respectively (Table 1), each of which could facilitate TG accumulation^8^,^10^. This severe nutritional limitation still allowed the cells to grow up to a 4.0-fold higher level than the initial one (see ‘0.0’ in Fig. 1c, cf., ‘initial’), however, the Chl content decreased to 54% of the initial level (data not shown), which suggested positive degradation of the PSI and/or PSII complexes. As to TL, its content became 1.5-fold higher than control (see ‘0.0’ in Fig. 1d, cf., ‘0.0’ in Fig. 1b). The increase of TL could be explained almost exclusively by TG, which accumulated to 117 μM as fatty acids in the culture (see ‘0.0’ in Fig. 1d) or 41.4 mol% relative to TL (see ‘0.0 in 1/400 GB5’ in Fig. 1e). Therefore, nutrient-limiting stress, cooperatively with hyperosmotic stress, could induce TG accumulation in the air-drying cells.

### Effects of combinatory stress of hyperosmosis and nutrient-limitation on the TG content

Both hyperosmosis and nutrient-limitation were then imposed simultaneously on *C. kessleri* cells (Figs 1c–e and 2c–e). The combination of sorbitol-induced hyperosmosis with nutrient-limitation, as compared with nutrient-limitation, tended to more seriously impair cell growth (Fig. 1c). Concomitantly, the combinatory stress with 0.3 or 0.6 M sorbitol had great effects to stimulate TL accumulation to >500 μM in the culture (Fig. 1d, cf., 295 μM in the culture of nutrient-limited cells). Concerning TG, 0.3 or 0.6 M sorbitol also led to pronounced induction of its accumulation (Fig. 1d,e: >300 μM in the culture and >60 mol% relative to TL). In particular, the combinatory stress with 0.3 M sorbitol gave the maximal accumulation level of TG in the culture (368 μM) with the high TG content relative to TL (61.9 mol%). A similar trend was observed in the case of NaCl, showing that the combinatory stress, relative to single nutrient-limiting stress, retarded cell growth more severely (Fig. 2c), and that the weak hyperosmotic stress with 0.15 M NaCl with the cooperation of nutrient-limitation remarkably elevated the induced level of TG accumulation (Fig. 2d,e).

**Figure 3:**
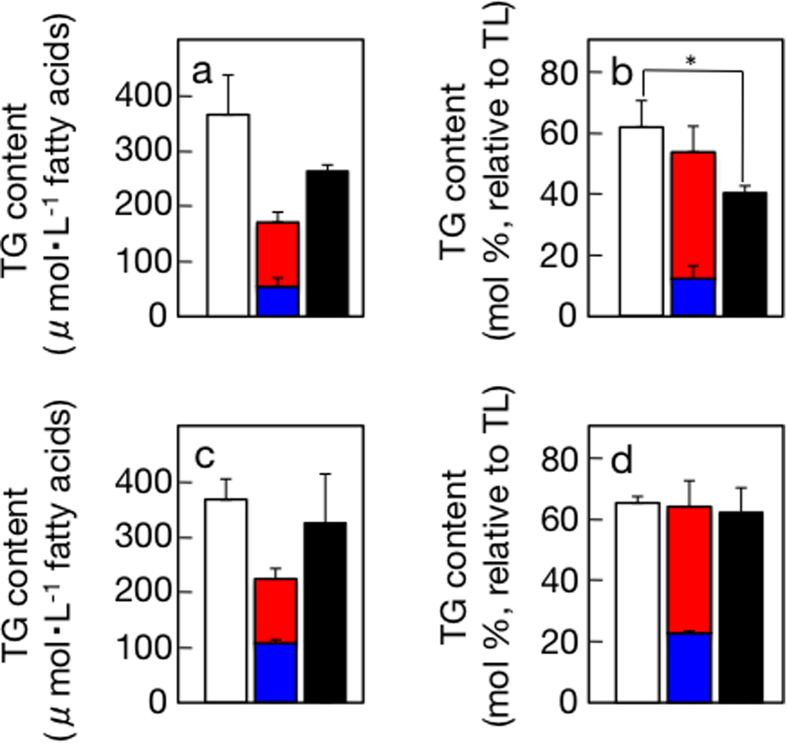
Additive or synergistic effects of hyperosmotic and nutrient-limiting stress on TG accumulation. (**a,b**) Open bars indicate TG contents in the cultures (**a**) or those relative to TL (**b**) under combinatory stress with 0.3 M sorbitol whereas red, blue, and closed bars indicate TG contents under single stress conditions with nutrient-limitation, 0.3 M sorbitol, and 0.6 M sorbitol, respectively. (**c,d**) Open bars indicate TG contents in the cultures (**c**) or those relative to TL (**d**) under combinatory stress with 0.15 M NaCl whereas red, blue, and closed bars indicate TG contents under single stress conditions with nutrient-limitation, 0.15 M NaCl, and 0.3 M NaCl, respectively. The values were from Figs 1b,d,e and 2b,d,e.

It was of note that the combination of 0.3 M sorbitol or 0.15 M NaCl with nutrient-limitation, respectively, showed a synergetic effect of the two discrete stresses on TG accumulation in the culture (Fig. 3a,c), whereas the combination exerted an additive effect on TG accumulation in TL (Fig. 3b,d). In particular, the effect of the combinatory stress of 0.3 M sorbitol and nutrient-limitation was greater than the effect of 0.6 M sorbitol, i.e., double stress with 0.3 M sorbitol, which indicated the superiority of the combinatory stress to intensification of the hyperosmotic stress (Fig. 3a,b). Meanwhile, the effect of the combinatory stress with 0.15 M NaCl and nutrient-limitation on TG accumulation in TL was similar to that of 0.3 M NaCl (Fig. 3d).

### Effects of hyperosmotic or nutrient-limiting stress on *C. reinhardtii* cells

**Figure 4:**
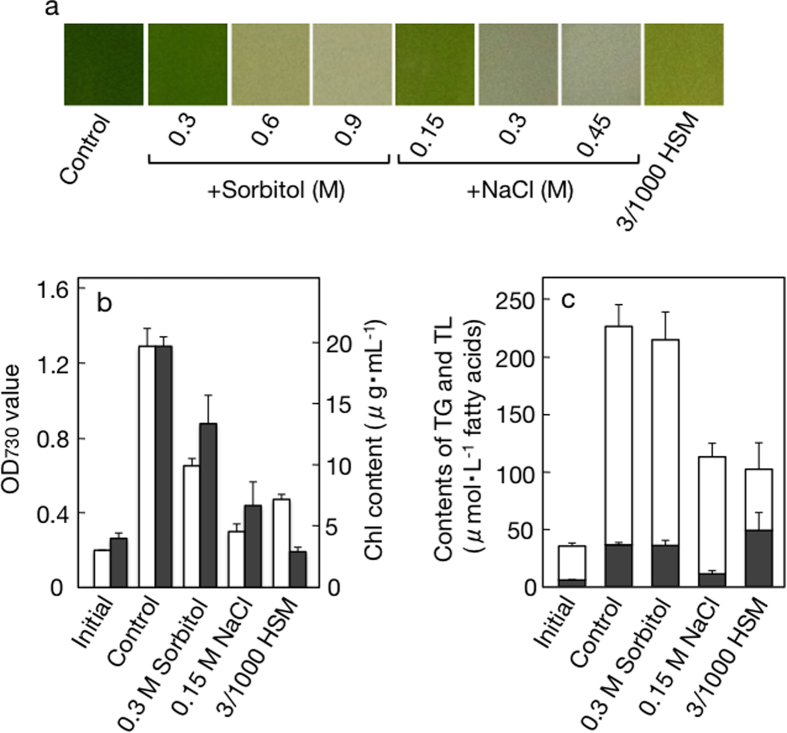
Effects of hyperosmotic or nutrient-limitation stress on cell growth and TG accumulation in *C. reinhardtii*. (**a**) Photograph of 2-day cultures under the respective conditions. (**b**) OD_730_ values (open bars) or Chl contents (closed bars). The initial levels of OD_730_ were adjusted to 0.2. (**c**) Accumulated levels of TG (closed bars) or TL (see top values of open bars) in the culture. The values shown are the averages ± SE for three distinct groups of data.

The impacts of hyperosmotic or nutrient-limiting stress were then investigated in *C. reinhardtii. C. reinhardtii*, distinct from *C. kessleri*, was highly sensitive to hyperosmotic stress such that ≧0.6 M sorbitol or ≧0.3M NaCl induced bleaching of Chl and resultant cell death (Fig. 4a). The weak hyperosmosis with 0.3 M sorbitol or 0.15 M NaCl allowed the cells to grow to only 51% and 23%, respectively, of the control level (Fig. 4b). In line with this, Chl accumulation was repressed to as low as 68% and 34% of the control levels in the sorbitol- and NaCl-stressed cells, respectively (Fig. 4b). This green alga when grown photoautotrophically, as compared with *C. kessleri*, showed a markedly higher cellular TG content, i.e., 16.4 mol%, relative to TL on the basis of fatty acids, even under normal growth conditions (Control in Fig. 4c). Accordantly, TG accumulated to 36 μM in the culture in two days under normal conditions, however, neither sorbitol nor NaCl stimulated TG accumulation (Fig. 4c). In contrast, in *C. reinhardtii* cells cultured in a 100-fold diluted medium, TG accumulated to 46.0 mol% relative to TL (Fig. 4c). This TG accumulation was accompanied by both retarded growth and Chl degradation as in the nutrient-limited cells of *C. kessleri* (Fig. 4b).

### Utilization of seawater for imposition of the combinatory stress in *C. kessleri*

**Figure 5:**
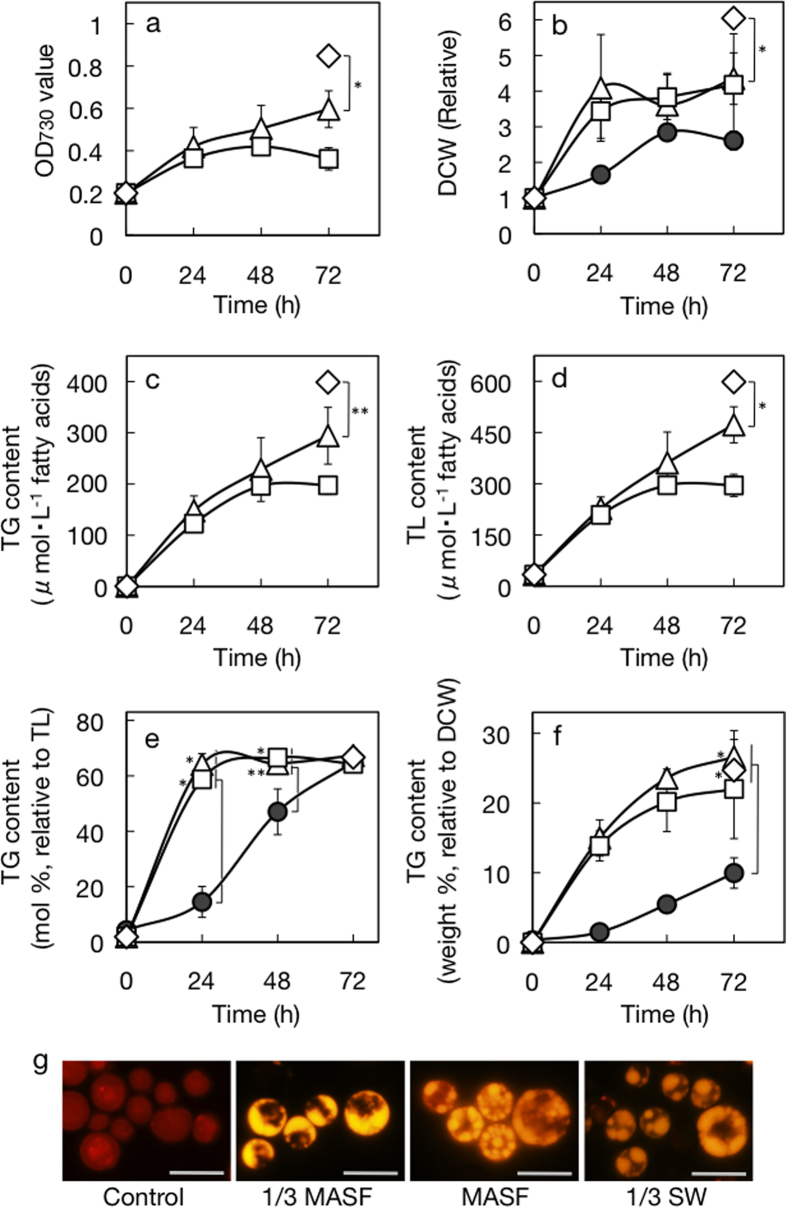
Effects of hyperosmotic stress, with the use of seawater, on cell growth and TG accumulation in *C. kessleri*. The cells were grown for 3 days in 1/3 MASF (triangles) or MASF (squares) medium, or 1/3 SW (diamonds), and then subjected to the following measurements. (**a**) OD_730_ values of the culture. The initial levels of OD_730_ were adjusted to 0.2. (**b**) Dry cell weights relative to the initial level. (**c**) TG contents in the culture. (**d**) TL contents in the cultures. TG contents relative to TL (**e**) or dry cell weight (**f**). Our previous data for the air-drying cells (closed circles) are also included in (**b,e,f**)^9^. The values in (**e,f**) were estimated from data of (**c,d**), and those of (**b,c**), respectively. The measurements were performed every other day for the cultures in 1/3 MASF or MASF medium, and once on Day 3 for the culture in 1/3 SW. (**g**) Nile-red stained lipid droplets in cells stressed with or without the use of seawater. White bars represent 10 μm. The values shown are the averages ± SE for three distinct groups of data. The significance of differences was evaluated by Student’s t test. *P < 0.05. **P < 0.1. In (**e,f**), see differences in the TG content between air-drying and seawater-stressed cells, at respective time points.

Artificial seawater, MASF, contains 0.45 M Na^+^ and 0.51 M Cl^−^ with a shortage of several nutrients. For induction of TG accumulation, we first utilized 1/3 MASF, which was similar to the combinatory stress medium with 0.15 M NaCl in NaCl concentration and severe limitation as to N-, P-, Fe-, Mn-, and Zn-sources (Table 1). Irrespective of whether on the basis of OD_730_ (Fig. 5a) or dry cell weight (DCW, Fig. 5b), a large part of cell growth was achieved in a day during culturing for three days in 1/3 MASF. The TG content showed an ongoing increase up to 294 μM in the culture, together with an increase in the TL content (Fig. 5c,d). The TG content relative to TL on a fatty acid basis abruptly increased to 64 mol% in a day, and thereafter remained at the increased level for the next two days (Fig. 5e). Meanwhile, the TG content in dry cells continuously increased for three days up to 26.6 weight% (Fig. 5f). Outstandingly, it took only two days for the TG content to exceed 20% of the biomass. In accordance with these results, Nile-red stained cells contained lipid droplets as a few large intracellular globules that emitted yellow fluorescence against the background of red autofluorescence of Chl under a fluorescence microscope (Fig. 5g). Non-diluted MASF, as compared with 1/3 MASF, led to a similar increasing pattern of the DCW (Fig. 5b). The cells achieved the maximal TG content relative to TL in a day in MASF as well as in 1/3 MASF, with lipid droplets greatly accumulating (Fig. 5e,g). However, MASF was found to be inferior to 1/3 MASF concerning the induction level of TG accumulation in the culture (Fig. 5c), compatible with the lower TG accumulation in the culture with the combinatory stress with 0.45 M NaCl than with 0.15 M NaCl (Fig. 2d).

The cells were then cultured for three days in authentic seawater diluted three-fold (1/3 SW) and showed more vigorous growth than in 1/3 MASF (Fig. 5a,b). Therefore, 1/3 SW had less deleterious effects on cellular physiological processes. The accumulated levels of TG with 1/3 SW, similar to those with 1/3 MASF, amounted to 24.7 weight% and 66.7 mol%, relative to biomass and TL, respectively (Fig. 5e,f). Importantly, the better growth in 1/3 SW was beneficial for accumulation of TG in the culture up to 399 μM (Fig. 5c). Overall, it turned out that the use of seawater, irrespective of whether it was artificial or authentic, led to a strong stimulatory effect on TG accumulation, consistent with results of the combinatory stress with 0.15 M NaCl (Fig. 2). The standard seawater, as compared with 1/3 MASF, includes less abundant N-sources, which itself seemed disadvantageous for *C. kessleri* cells to grow (Table 1). Explanation of the contrary effects of 1/3 SW waits for elucidation of the actual chemical compositions of the seawater used in this study.

## Discussion

This study led to three discoveries through investigation of stressors that are responsible for induction of TG accumulation in air-drying *C. kessleri* cells^9^. First of all, it was proved with the use of two different solutes, sorbitol and NaCl, that environmental hyperosmosis is a novel stressor for induction of TG accumulation (Figs 1b,e and 2b,e). Also of note was the more prominent effect of NaCl than that of sorbitol at the same hyperosmotic level: most strikingly, 0.45 M NaCl, comparable to N-starvation or air-drying^4^, induced TG accumulation to as high as 75.3 mol% as to TL, while 0.9 M sorbitol increased it to merely 48.5 mol% (Figs 1e and 2e). NaCl is known to perturb intracellular ionic homeostasis through flux of Na^+^ and Cl^−^ into the cells^14^,^15^,^16^, and, in *C. reinhardtii*, to induce TG accumulation at low concentrations (0.02 to 0.1 M), which was evidently unrelated to hyperosmosis^7^. In our study, it is likely that influx of Na^+^ and Cl^−^ into the NaCl-stressed cells perturbed ionic homeostasis and, in addition, elevated intracellular osmotic pressure to a comparable level to that of sorbitol-stressed cells. This ionic perturbation might contribute to a greater impact on TG accumulation in NaCl-stressed cells than in sorbitol-stressed ones. The TG content in TL with 0.3 M NaCl was much higher than that with 0.6 M sorbitol, being no way inferior to that with the combinatory stress of nutrient-limitation and 0.15 M NaCl (Fig. 3b,d). This greater effect of 0.3 M NaCl would also be explained by additive induction of TG accumulation by the ionic perturbation. Some research groups demonstrated that NaCl at high concentrations induced TG accumulation in algal cells with retardation of their growth^17^. However, two factors of the NaCl stress, i.e., hyperosmosis and ionic perturbation, were not experimentally evaluated in these studies, the factors responsible for TG accumulation in the NaCl-stressed cells having never been determined.

Meanwhile, it was of note that weak or moderate hyperosmosis could activate polar lipid synthesis. NaCl-induced increases in the contents of membrane lipids have been reported in some algal cells including those of *Cladophora vagabunda*^18^ or also in cell suspension of a seed plant, *Catharanthus roseus*^19^. It will be investigated in the future how and why *C. kessleri* cells modulate biogenesis of membrane systems including that of thylakoids under hyperosmotic conditions.

The accumulation level of TG relative to TL, which would indicate how preferentially metabolic carbon flows into TG synthesis, relative to the other lipid metabolism, depends on the environmental conditions for cell growth in an alga, as observed in Figs 1e, 2e, and 5e, and also on algal species if under the same environmental conditions: e.g., the maximal TG content in TL is higher in N-starved cells of *C. kessleri* than in S-starved ones of it, and *C. kessleri* surpassed *C. reinhardtii* in the content under the respective conditions^10^. We recently found that the *SAC1* and *SNRK2.2* genes are involved in setting of the maximal level of TG accumulation in S-starved cells of *C. reinhardtii* through their positive and negative regulation, respectively, as to the expression levels of the genes for TG synthesis^10^. Setting of the maximal levels would conversely help ensure membrane lipids in quantity, including thylakoid ones, for proper functioning of the membranes under respective aberrant conditions, and inevitably for ongoing TG synthesis (e.g., Fig. 5c). An upper limit concerning TG accumulation in TL seemed to be intrinsically set at around 70 mol% in *C. kessleri*, as could be achieved in cells on exposure to 0.45 M NaCl (Fig. 2e), air-drying or N-starvation^9^,^10^.

Second, limited availability of all nutrients is effective for TG accumulation in both *C. kessleri* and *C. reinhardtii* (Figs 1d,e and 4c). Information has began to be collected for green algae concerning whether or not single depletion of nutrients has a positive effect on TG accumulation^8^,^20^,^21^. In this study, despite possible damage to extensive physiological aspects, algal cells limited in all nutrients were found to be so tough as to show growth up to a 4.0-fold level relative to the initial one, and concomitant accumulation of TG to a level comparable to that in S-starved cells^10^. Besides TG, polar lipids that constitute membrane systems were quantitatively increased under all-nutrient limited conditions (Fig. 1d, see 0.0, cf., initial). The continued biogenesis of the membranes and probable accumulation of lipid bodies filled with TG would be consistent with the increase in the OD_730_ value (Fig. 1c). The increased level in cell growth was similar to that observed for *C. kessleri* (data not shown) and other algal species^22^ under N-starved conditions. The simultaneous facilitation of TG accumulation would be explained by limited inclusion of N-, S-, and Fe-sources, at least in the medium^8^,^10^. The results were compatible with a previous report by Li *et al*.^23^, who showed induced accumulation of TL and lipid droplets in *C. kessleri* cells cultured in a 10-fold diluted medium, however, no quantification of TG was performed. Again, we could demonstrate the effectiveness of limitation of all nutrients on TG accumulation for the first time, and, in this context, propose the necessity to reevaluate the effects of nutritient-limitation on TG accumulation, not only as to single ones, but also to combinations of nutrients including all of them.

Cells of *C. kessleri* or *C. reinhardtii* with all nutrients including N- and S-sources limited should be repressed as to global protein synthesis. Extremely, it seemed that the complexes of photosystems I and II were markedly degraded in these cells, in view of a decrease of Chl to ca. 50% of the initial level. We recently proposed that S- or N-starvation induces TG accumulation in *C. reinhardtii* cells through diversion of carbon metabolic flow from the synthesis of proteins to that of carbon-storage compounds like TG as well as through regulation of expression of the genes responsible for TG synthesis in a manner specific to the nutrients depleted^10^. A similar change in metabolic carbon-flow, together with possible induction of the genes for TG synthesis, would contribute to TG accumulation in nutrient-limited cells of this study.

*C. kessleri* cells stressed with 0.6 M sorbitol, similar to those with nutrient-limitation, showed TG accumulation to ca. 40 mol% in TL (Fig. 1e). It was likely that protein synthesis including that of photosystem complexes was less severely impaired in the 0.6 M sorbitol-stressed cells than in nutrient-limited cells (ca. a 3-fold increase of Chl from the initial level in the sobitol-stressed cells, Fig. 1a, cf., a decrease of Chl by ca. 50% in the nutrient-limited cells), therefore induction mechanism of the sorbitol-stressed cells for TG accumulation might not so greatly depend on the change in metabolic carbon flow. Freezing stress generally causes intracellular dehydration, which is initiated by extracellular ice formation^24^. In a seed plant, *Arabidopsis thaliana*, upon exposure to freezing stress, MGDG is metabolized to oligogalactolipids and diacylglycerol, the latter of which is further acylated for the synthesis of TG, and thereby is sequestered from the membranes^25^. This remodeling of lipids was considered to be adaptation mechanism for this severe environment, since non-bilayer lipids, MGDG and DG, could otherwise perturb membrane stability^25^. It should be investigated in the future whether or not similar remodeling of lipids is involved in the mechanism for TG accumulation in hyperosmotically stressed cells of *C. kessleri*.

Third, our study was the first to demonstrate the effective cooperation of two quite discrete stressors, weak hyperosmosis and nutrient-limitation, for TG accumulation (Fig. 3). Above all, this effect could be reproduced with the use of 3-fold diluted seawater (Fig. 5). Moderate or strong hyperosmosis, distinct from weak hyperosmosis, had only smaller effects as to facilitation of TG accumulation in the culture when combined with nutrient-limitation (Figs 1d and 2d), probably because of too severe environment for the cells to cope with. Meanwhile, the level of algal TG production depends on how preferentially the metabolic flow of carbon enters lipid metabolism, relative to the other carbon metabolism, and how exclusively the carbon flow in the lipid metabolism is directed to TG synthesis, as described above. Also important is how high the cell growth attained is. The combinatory stress, e.g., that with 1/3 MASF, fulfilled these requirements so well as to allow a preferential increase of TG in biomass, accumulation of TG up to >60 mol% in TL, i.e., to a level close to an intrinsic upper limit (see above), and a persistent increase of TL in the culture (Fig. 5d–f). In this context, it was of interest that, in cells cultured in 1/3 MASF, the TG content relative to TL increased to a plateau level of 64.1 mol% in a day, while its content in biomass or that in the culture continued to increase (Fig. 5d–f). These results implied that a system that causes preferential carbon-flow into TG synthesis, relative to the other lipid metabolism, could be established much sooner than a system that diverts carbon-flow more greatly into the lipid metabolism, relative to the other carbon metabolism. In the future, it will be investigated in *C. kessleri* cells what the metabolic and molecular basis of the combinatory stress-induced TG accumulation is, including that which exerts the additive and synergetic effects, and also what kind of physiological roles it plays.

On the other hand, the similar plateau level in DCW for the cells in 1/3 MASF and those in MASF might be explained by their distinct metabolic states. The severer defect in nutrients in 1/3 MASF, as compared with that in MASF, caused the cells to store more abundant storage compounds such as TG (Fig. 5c), probably at the expense of the synthesis of fundamental cellular components such as proteins. It is possible such balancing of metabolism resulted in the similar level of DCW, which, however, needs experimental verification in the future.

Air-dying cells of *C. kessleri* placed on a glass fibre membrane for TG accumulation were subjected to gradual dehydration until the water content of the membrane including cells was reduced to 50% of the initial level in four days^9^. It was probable that the cells were harder to dehydrate than the glass fibre membrane itself, retaining much more than 50% of the initial intracellular water level. Assuming that the normal cellular osmolarity in *C. kessleri* is equal to that in *Chlorella emersonii* (0.24 osm/L)^26^, we can estimate that the osmotic pressure inside air-drying cells reached a level far below the one that is caused by 0.48 M sorbitol or 0.24 M NaCl. We thus consider that progression of cellular dehydration at a certain weak level under nutrient-limited conditions is the key for the success in TG accumulation in air-drying cells of *C. kessleri*. Meanwhile, despite the common involvement of these two stressors in induction of TG accumulation, the combinatory stress was definitely superior to the air-drying one in the rate and/or final level of TG accumulation (Fig. 5e,f). The superiority of the combinatory stress could be attributed to illumination with light at a higher intensity (78.6 μmol photons·m^−2^·s^−1^, cf., 15 μmol photons·m^−2^·s^−1^ for the air-drying stresses), which should promote photosynthesis for TG accumulation in liquid-culturing cells, but is toxic to air-drying cells (data not shown).

Besides *C. kessleri*, a group of green algae are classified as oleaginous species, including *C. pyrenoidosa*^27^, *S. obliquus*^28^, *Lobosphaera incia*^29^, and *Chlorella protothecoides*^30^. These species contain high-value polyunsaturated fatty acids such as linoleic, linolenic and/or arachidonic acids. Moreover, determination of genomic DNA sequences and/or transformation by gene introduction has been successful for some species, which will offer a platform for future improvement of their TG in quality and quantity^31^,^32^,^33^. These oleaginous green algal species, despite having the above merits, exhibit slow cell growth under TG-accumulating conditions, which will be an obstacle for their utilization for industrial TG production: e.g., in *C. pyrenoidosa*, it took as long as 7 days for TG to accumulate to the maximal level (34% of biomass) under N-starved conditions^27^. The combinatory stress in our culturing system could trigger a short-term response of relatively great accumulation of TG in *C. kessleri* cells (only 2 and 3 days for TG accumulation to 24 and 27 weight%, respectively). Above all, it is economically attractive that the culture medium for induction of TG accumulation can be simply diluted seawater, which is available at a low cost. Therefore, development of a culturing system for combinatory stress should be one of the promising options for industrialization of green algal TG production. Meanwhile, our study also showed that the growth of *C. reinhardtii* cells was highly sensitive to hyperosmotic environments, which was consistent with previously reported intolerance of photosynthesis to hyperosmotic stress in this alga^34^, and that its cells were unable to increase the TG content in response even to weak hyperosmosis (Fig. 4). We therefore considered that the combinatory stress would be much less effective for TG accumulation in *C. reinhardtii* than in *C. kessleri*. Consistent with this thought, less effective induction of TG accumulation was previously observed under air-drying conditions in *C. reinhardtii* than in *C. kessleri*^9^. The system for combinatory stress thus demands appropriate selection of algal species dependent not only on industrial usage, but also on a high ability to tolerate 0.15 M NaCl. Elucidation of physiological properties that allow *C. kessleri* cells to withstand hyperosmotic stress remains for future study.

In conclusion, this study demonstrated that hyperosmosis is a novel stressor for induction of TG accumulation in *C. kessleri*, with the greatest effect of 0.45 M NaCl causing TG accumulation to 75.3 mol% in TL, i.e., a level comparable to that induced by N-starvation. Moreover, it turned out that even the weak hyperosmosis with 0.15 M NaCl or 0.3 M sorbitol when combined with nutrient-limitation most strongly stimulate TG accumulation in the culture. These findings led us to deduce that air-drying stress induces TG accumulation through the action of combinatory stress, and most importantly substantiated the usefulness of 1/3 diluted SW as a medium for combinatory stress for algal TG production.

## Material and Methods

### Algal strains and growth conditions for their cells

The freshwater algal strains used were *C. kessleri* 11 h and *C. reinhardtii* 137c^35^,^36^. The culture medium for normal growth was prepared through 4-fold dilution of Gamborg’s B5 medium (Wako Pure Chemical Industries, Osaka; 1/4 GB5) for *C. kessleri* or 3/10 HSM for *C. reinhardtii*^19^,^37^,^38^. Each medium contained 0.3, 0.6 and 0.9 M sorbitol, or 0.15, 0.3, and 0.45 M NaCl for imposition of hyperosmotic stress, whereas it was 100-fold diluted (1/400 GB5 or 3/1000 HSM) for limitation stress as to all nutrients. The combinatory stress of hyperosmosis and nutrient-limitation was imposed on cells in the diluted medium with a corresponding concentration of sorbitol or NaCl. Marine Art SF-1 (Osaka-yakken, Osaka, Japan) supplemented with 51 nM phosphate (MASF) was used as artificial seawater to match authentic seawater (SW) in the amount of phosphorus. SW was obtained at Yuiga-hama beach at Kamakura city in Japan. Three-fold diluted MASF and SW (1/3 MASF and 1/3 SW, respectively), or MASF was used as the culture medium for imposition of the combinatory stress with seawater.

The OD_730_ values of cultures were determined for monitoring of cell growth. Chl was quantitated by spectroscopy after extraction of it from cells with 100% methanol, as describe by Sato *et al*.^36^. A spectrophotometer, DU640 (Beckman, USA), was used for monitoring of the cell growth and Chl content. The cells are pre-cultured normally at 30 °C with aeration in an oblong glass vessel under illumination (78.6 μmol photons·m^−2^·s^−1^) until the OD_730_ value became ca. 0.5. The pre-cultured cells were harvested by centrifugation, and then were resuspended in a medium corresponding to the stress to be imposed with adjustment of the OD_730_ value to 0.2 or 0.3. The cells were then cultured for two days under the respective conditions for hyperosmosis or nutrient-limitation, or their combination, or for three days in 1/3 MASF, MASF or 1/3 SW.

### Extraction and analysis of lipids

The stressed cells were harvested by centrifugation, and then used for extraction of TL according to the method of Bligh and Dyer^39^. TG was separated from the other lipid classes by TLC on precoated silica gel plates [Merck 5721] with a solvent system of hexane/diethylether/acetate (70:30:1, v/v/v). The TG spot was visualized through illumination with UV light after spraying with primuline (0.01% in 80% acetone, w/v), and the silica gel containing TG was then scraped off. Fatty acid methyl esters were prepared from TL and TG by heating at 95 °C with 5% anhydrous methanolic HCl for subsequent quantitative analysis as to their constituent fatty acids by capillary GLC, as described previously^36^. The fatty acid content of each fraction was estimated with arachidonic acid as an internal standard.

### Microscopic observation of lipid droplets

A culture of algal cells was centrifuged to pellet the cells, which were then suspended in 100 mM DMSO. A Nile red solution (0.5 mg/mL in acetone) was added to the cell suspension (1:50, v/v), and the stained cells were observed under a fluorescence microscope (BX-FLA; Olympus Optical Co., Tokyo, Japan) with the use of a 460–490 nm excitation filter.

## Additional Information

**How to cite this article**: Hirai, K. *et al*. Hyperosmosis and its combination with nutrient-limitation are novel environmental stressors for induction of triacylglycerol accumulation in cells of *Chlorella kessleri. Sci. Rep.* **6**, 25825; doi: 10.1038/srep25825 (2016).

## Figures and Tables

**Table 1:** Chemical compositions of respective culture media for the combinatory stress.

	0.15M NaCl in 1/400 GB5	1/3 MASF^a^	1/3 SW^b^
NO_3_^−^ or NH_4_^+^	6.7 × 10^−2^	2.9 × 10^−5^	1.7 × 10^−6^
HPO_4_^2−^	2.7 × 10^−3^	1.7 × 10^−5^	1.7 × 10^−5^
SO_4_^2−^	5.5 × 10^−3^	9.2	9.3
Na^+^	1.5 × 10^2^	1.5 × 10^2^	1.6 × 10^2^
Cl^−^	1.5 × 10^2^	1.7 × 10^2^	1.8 × 10^2^
K^+^	6.2 × 10^−2^	3.0	3.3
Ca^2+^	2.6 × 10^−3^	3.4	3.3
Mg^2+^	2.5 × 10^−3^	1.6 × 10	1.8 × 10
Fe(OH)_3_	2.5 × 10^−4^	6.2 × 10^−6^	6.7 × 10^−8^
Mn^2+^	1.7 × 10^−4^	1.0 × 10^−6^	6.7 × 10^−7^
B(OH)_3_	1.2 × 10^−4^		1.4 × 10^−1^
Zn^2+^	1.7 × 10^−5^		2.7 × 10^−7^
IO_3_^−^	1.1 × 10^−5^	1.6 × 10^−4^	1.2 × 10^−4^
MoO_4_^2−^	2.6 × 10^−6^	4.9 × 10^−6^	3.5 × 10^−5^
Co^2+^	2.6 × 10^−7^	2.8 × 10^−6^	4.0 × 10^−8^
Cu^2+^	2.5 × 10^−7^		4.3 × 10^−7^
HCO_3_^−^		7.5 × 10^−1^	6.7 × 10^−1^
Br^−^		2.7 × 10^−1^	2.8 × 10^−1^
Sr^2+^		2.7 × 10^−2^	3.0 × 10^−2^
F^+^		2.4 × 10^−2^	2.3 × 10^−2^
Al(OH)_3_		1.1 × 10^−5^	1.7 × 10^−6^
Li^+^		7.9 × 10^−6^	8.7 × 10^−3^
WO_4_^2−^		2.0 × 10^−6^	1.7 × 10^−8^
Total solutes	3.0 × 10^2^	3.5 × 10^2^	3.7 × 10^2^

^a,b^Chemical compositions of 1/3 MASF and 1/3 SW were estimated on the basis of data from manufacture’s instructions (Osaka-yakken, Osaka) and those from chronological scientific tables (Maruzen, Tokyo), respectively. The values are indicated at mM.
